# Genomic Instability and Telomere Fusion of Canine Osteosarcoma Cells

**DOI:** 10.1371/journal.pone.0043355

**Published:** 2012-08-16

**Authors:** Junko Maeda, Charles R. Yurkon, Hiroshi Fujisawa, Masami Kaneko, Stefan C. Genet, Erica J. Roybal, Garrett W. Rota, Ethan R. Saffer, Barbara J. Rose, William H. Hanneman, Douglas H. Thamm, Takamitsu A. Kato

**Affiliations:** 1 Department of Environmental & Radiological Health Sciences, Colorado State University, Fort Collins, Colorado, United States of America; 2 Department of Clinical Sciences, Colorado State University, Fort Collins, Colorado, United States of America; Bauer Research Foundation, United States of America

## Abstract

Canine osteosarcoma (OSA) is known to present with highly variable and chaotic karyotypes, including hypodiploidy, hyperdiploidy, and increased numbers of metacentric chromosomes. The spectrum of genomic instabilities in canine OSA has significantly augmented the difficulty in clearly defining the biological and clinical significance of the observed cytogenetic abnormalities. In this study, eight canine OSA cell lines were used to investigate telomere fusions by fluorescence *in situ* hybridization (FISH) using a peptide nucleotide acid probe. We characterized each cell line by classical cytogenetic studies and cellular phenotypes including telomere associated factors and then evaluated correlations from this data. All eight canine OSA cell lines displayed increased abnormal metacentric chromosomes and exhibited numerous telomere fusions and interstitial telomeric signals. Also, as evidence of unstable telomeres, colocalization of γ-H2AX and telomere signals in interphase cells was observed. Each cell line was characterized by a combination of data representing cellular doubling time, DNA content, chromosome number, metacentric chromosome frequency, telomere signal level, cellular radiosensitivity, and DNA-PKcs protein expression level. We have also studied primary cultures from 10 spontaneous canine OSAs. Based on the observation of telomere aberrations in those primary cell cultures, we are reasonably certain that our observations in cell lines are not an artifact of prolonged culture. A correlation between telomere fusions and the other characteristics analyzed in our study could not be identified. However, it is important to note that all of the canine OSA samples exhibiting telomere fusion utilized in our study were telomerase positive. Pending further research regarding telomerase negative canine OSA cell lines, our findings may suggest telomere fusions can potentially serve as a novel marker for canine OSA.

## Introduction

Osteosarcoma (OSA) is the most prevalent bone cancer in dogs and humans [1], [2]. Aggressive behavior and frequent pulmonary metastasis characterize this cancer, making it difficult to treat and often fatal for diagnosed patients [3]. The standard treatment for OSA in both species has traditionally been amputation or limb-sparing surgery combined with chemotherapy [4]. Despite improvements in these treatments, 72% of dogs die as a result of metastasis within two years of diagnosis [5]. Due to the high mortality rate related to OSA, new and more effective treatment strategies such as molecular targeted therapy are necessary to render improved prognosis in canine patients with OSA. Additionally, canine OSA potentially serves as an important model for human OSA due to remarkable similarities [6]. Canine OSA displays striking resemblance to that of human OSA in tumor biology and behavior, including metastatic propensity [4]. Additionally, the incidence of spontaneous disease in canine populations is approximately ten times higher than that of humans [1], [7]. Furthermore, OSA progression rate in dogs usually exceeds the typical rate observed in humans, which allows rapid accrual of data for analysis [8]. Until recently, research in canine cancer models has been limited due to the relative lack of species-specific investigational tools [4]. As more canine specific tools become available, canine OSA shows promise as a model for therapeutic developments relating to human OSA [9], [10].

Chromosomal instabilities are hallmarks of most solid tumors in humans [11]. The normal canine karyotype is composed of 38 pairs of acrocentric autosomes and two metacentric sex chromosomes [12], [13]. Canine OSA presents with highly variable and chaotic karyotypes, including hypodiploidy, hyperploidy, and increased numbers of metacentric chromosomes [14]. Chromosomal instabilities may result from defective chromosomal segregation during mitosis, which can occur through several mechanisms including telomere dysfunction, centrosome amplification, dysfunctional centromeres, or defective spindle check-point control [15], [16]. The varied and often chaotic observed chromosomal abnormalities in canine OSA have significantly augmented the difficulty in clearly defining the biological and clinical significance of these cytogenetic abnormalities. Recent work has shown that OSA displays lower telomerase positivity relative to many other tumors [17]. While 85% of human tumors and 92–95% of canine tumors express telomerase, only 32–44% of human OSA and 73% of canine OSA are telomerase positive [18], [19], [20], [21], [22]. Telomeres, catalyzed by telomerase, are the nucleoprotein structures that cap the ends of linear chromosomes. In normal somatic cells, telomeres shorten with each cell cycle causing cell senescence and apoptosis [23]. Cancer cells possessing the ability to bypass telomere-induced senescence must have a mechanism by which telomeres are maintained. In the vast majority of human and canine cancers (>85%), this is achieved by reactivation of the enzyme telomerase, which synthesizes telomeric DNA [24], [25]. Some human tumor types that are telomerase independent can maintain their telomeres by an alternative mechanism known as alternative lengthening of telomeres (ALT) [26]. The principle functions of the telomere cap include prohibiting chromosome ends from re-joining and preventing the interpretation of damaged DNA as double-strand breaks (DSBs) which results in genomic instability and the activation of DNA damage checkpoints that signal cell cycle arrest or induce apoptosis [27], [28].

Telomere dysfunction resulting from eroded or unprotected telomere structures can lead to telomere fusion [29], [30]. In subsequent cell divisions, telomere fusion can trigger cycles of anaphase-bridging, breakage, and fusion that can lead to genomic arrangements of the type reflective of those frequently found in cancer [31]. In addition, effective mammalian telomeres require protein DNA-dependent protein kinase (DNA-PK), composed of a catalytic subunit (DNA-PKcs) and heterodimeric regulatory subunit (Ku70/Ku80) [32]. Deficiencies in DNA-PKs also cause dysfunctional telomeres, which can lead to telomere fusion [33]. In a previous study, telomeric fusions and shortening were observed in cells from a canine mammary pleomorphic adenoma transfected using a plasmid containing SV40 which is known to cause a higher proliferative capacity [34]. Known physical interactions between documented chromosomal fusions and telomeres prompted a detailed analysis of telomere status in canine OSA cell lines. In our study, we further investigated telomere fusion by focusing on the remarkable chromosome instabilities in canine OSA cell lines and primary cell cultures. Eight canine OSA cell lines were characterized by cytogenetic chromosomal studies pertaining to growth rates, radiosensitivity, telomerase activity, double-strand breaks, and presence of the telomere maintenance protein DNA-PKcs. Furthermore, we attempted to characterize our cell lines via correlations between each of the cytogenetic studies and chromosomal aberrations.

## Results

### Chromosome Abnormality, Cellular Doubling Times, and Cellular Radiosensitivity in Canine OSA

**Figure 1:**
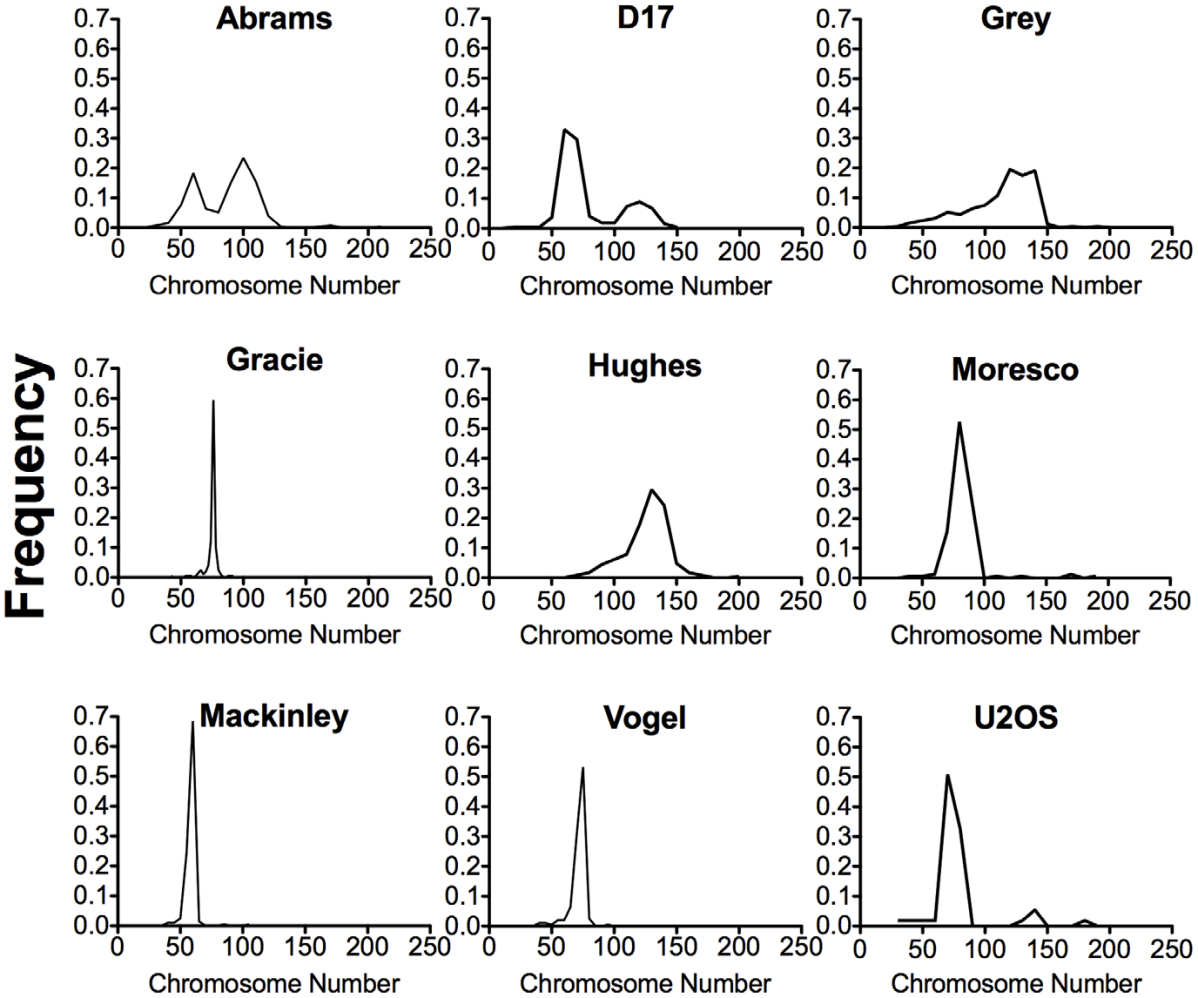
Distribution of chromosome number in the eight canine OSA cell lines and one human OSA cell line, U2OS. The data given is derived from the analysis of at least 150 metaphase chromosomes. Chromosome modes for the canine OSA cell lines are as follows; Abrams: (60, 100), D17: (60, 120), Grey: (120), Gracie: (75), Hughes: (130), Moresco: (80), MacKinley: (60), Vogel: (75), and U2OS: (70).

We characterized eight canine OSA cell lines with a cell proliferation assay and a classical cytogenetic assay (Table 1). Wide ranges of chromosome numbers were seen in Abrams and D17, and bimodal peaks were observed. The cell lines Grey, Hughes and Moresco displayed relatively large average numbers of chromosomes, with modal numbers of 120, 130, and 80 respectively. Alternatively, Gracie, MacKinley and Vogel had stable numbers of chromosomes with smaller averages. The frequency and distribution of chromosome numbers in hypodiploidy (less than 78 chromosomes) and hyperploidy (more than 78 chromosomes) are presented in Figure 1. All canine OSA cell lines showed increased numbers of metacentric chromosomes resulting from centric fusion events. Frequencies of metacentric chromosomes were 24% for Abrams, 55% for D17, 13% for Grey, 6% for Gracie, 14% for Hughes, 38% for Moresco, 27% for MacKinley, and 12% for Vogel.

**Table 1:** Characteristics in eight canine OSA cell lines.

OSA Cell Line	No. ofChromosomes*	No. of Metacentric Chromosomes**	Cell Doubling Time (hours)	Radiosensitivity(SF2)***	Ploidy Pattern byFlow Cytometry
Abrams	85.8±25.2	20.9±8.6	27.2	0.65	Triploid
D 17	79.1±25.4	43.2±14.2	22.2	0.70	Triploid
Grey	112.1±26.8	14.8±5.1	18.1	0.21	Tetraploid
Gracie	74.7±4.8	4.3±2.5	19.2	0.84	Diploid
Hughes	126.0±18.0	18.2±3.4	17.9	0.07	Tetraploid
Moresco	82.4±15.9	30.9±4.9	22.1	0.81	Triploid
MacKinley	58.2±5.1	15.7±3.2	27.6	0.49	Diploid
Vogel	71.7±6.3	8.5±2.9	35.9	0.22	Diploid

* Mean ± SD of chromosome number per cell from more than 150 metaphases.

** Mean ± SD of metacentrics per cell from more than 75 metaphases.

*** SF2: The survival fraction after 2 Gy. Calculated by Graph Pad Prism 5 with linear or linear quadratic regression.

**Figure 2:**
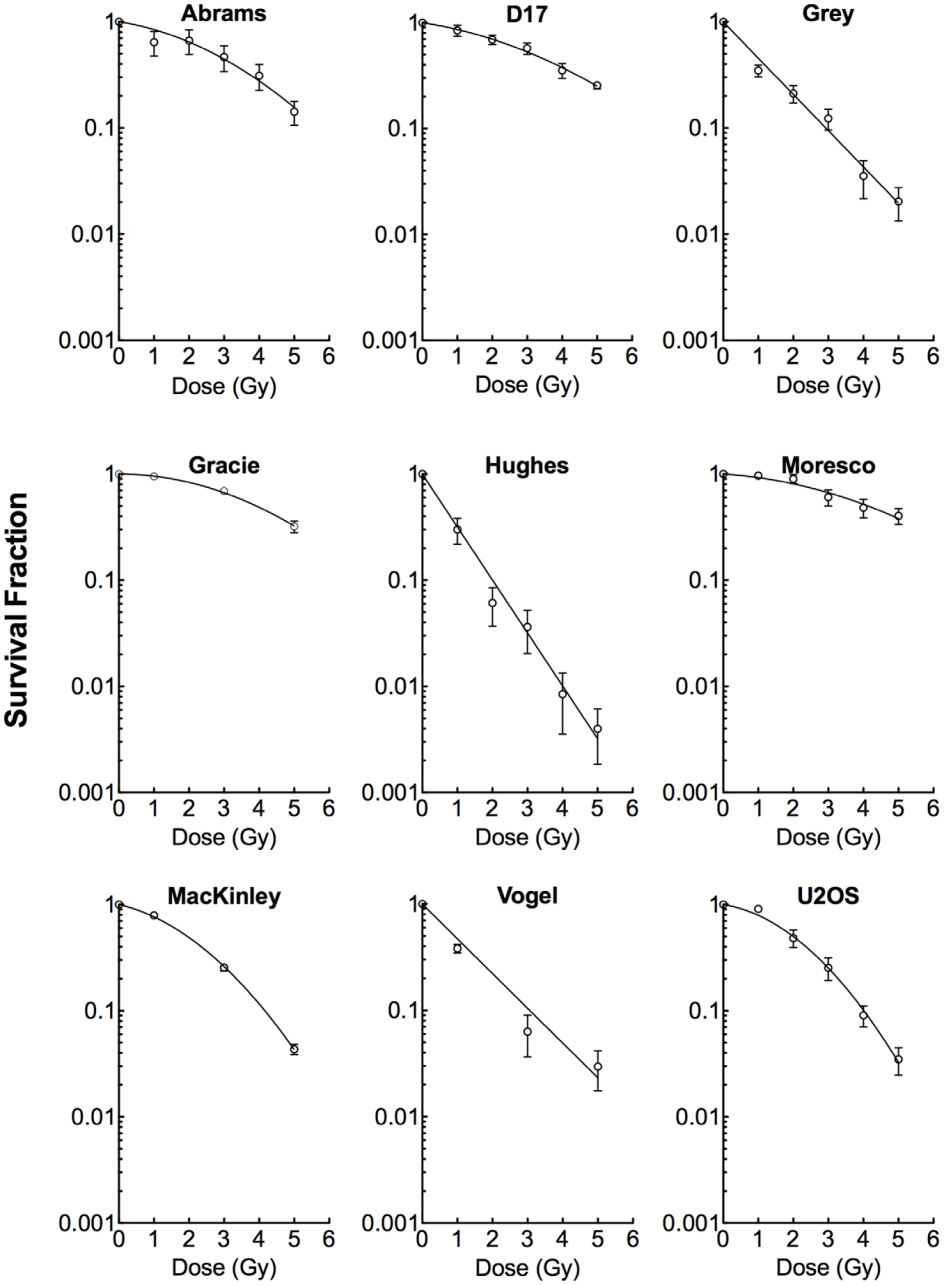
Radiation induced survival curves in eight canine OSA cell lines and one human OSA cell line, U2OS. Experiments were carried out at least three times and error bars indicate the standard error of the means.

The doubling times were approximately 36 hours for Vogel, 27 hours for Abrams and MacKinley, 22 hours for D17 and Moresco, and 18 hours for Grey, Gracie and Hughes. Radionsensitivities among the eight canine OSA cell lines were not uniform (Figure 2). Abrams, D17, Gracie and Moresco were quite resistant to ionizing radiation, while Grey, Hughes, MacKinley and Vogel were relatively more radiosensitive. The SF2 values (survival fraction after 2 Gy) of each cell line are summarized into Table 1. We determined the DNA content of the cell populations by flow cytometry (Table 1). A comparison between flow cytometry and chromosome number distribution by a metaphase analysis was made, and we found a relationship between abnormal ploidy and increased chromosome numbers.

### Telomere Fusions in Canine OSA

**Figure 3:**
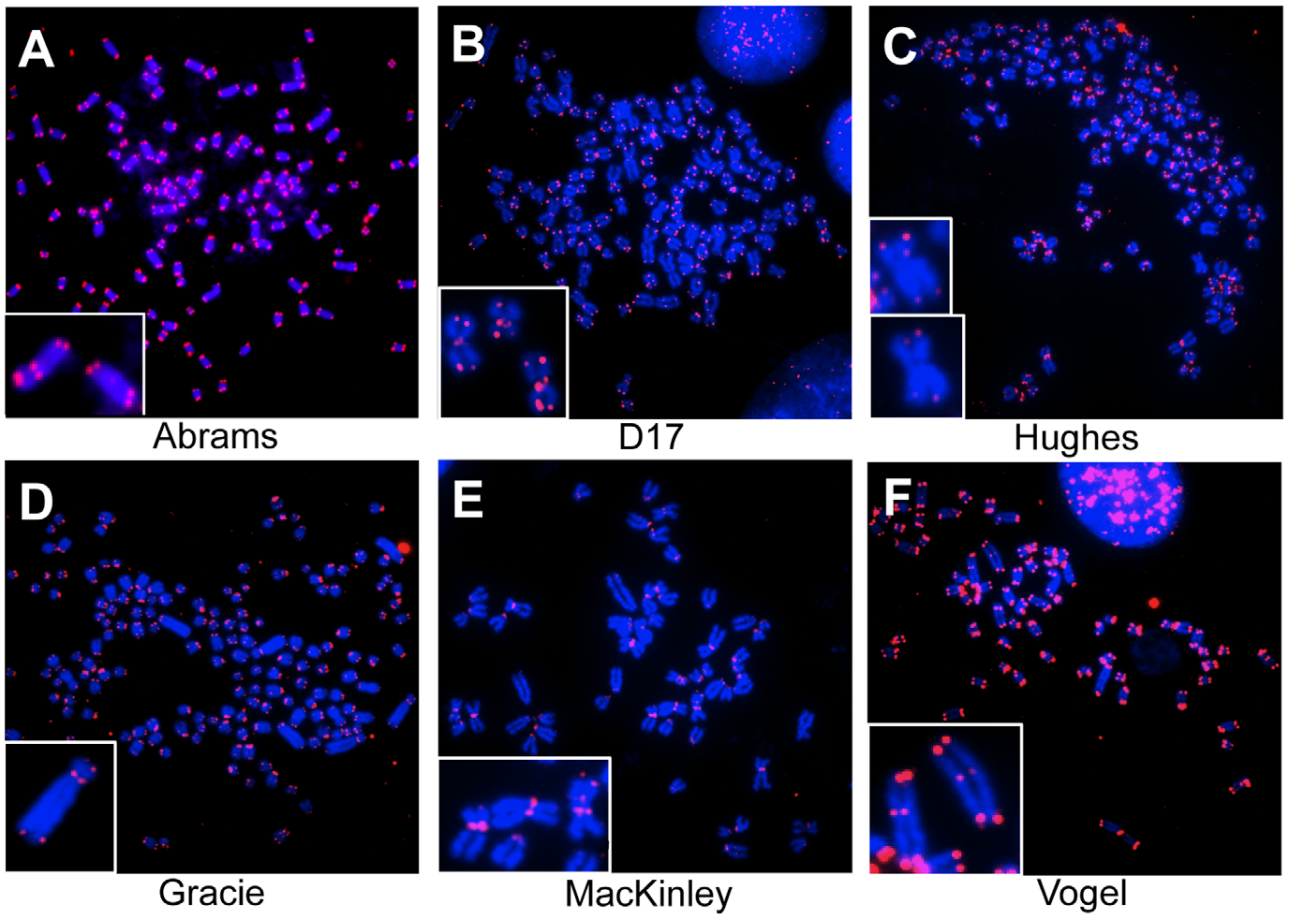
Telomere abnormalities. Representative FISH images of the eight canine OSA cell lines’ metaphase chromosomes hybridized with probes against telomeres. Blue represents DNA staining by DAPI and red represents a telomere signal by Cy3. Note the abnormal telomere signals in the magnification box; interstitial telomere signals (A and F), more than one telomere signal in centromere regions (B, D and E), and one or no telomere signal (C) is observed. Note that at the end of chromosomes, there is no telomere signal present (B and E).

**Figure 4:**
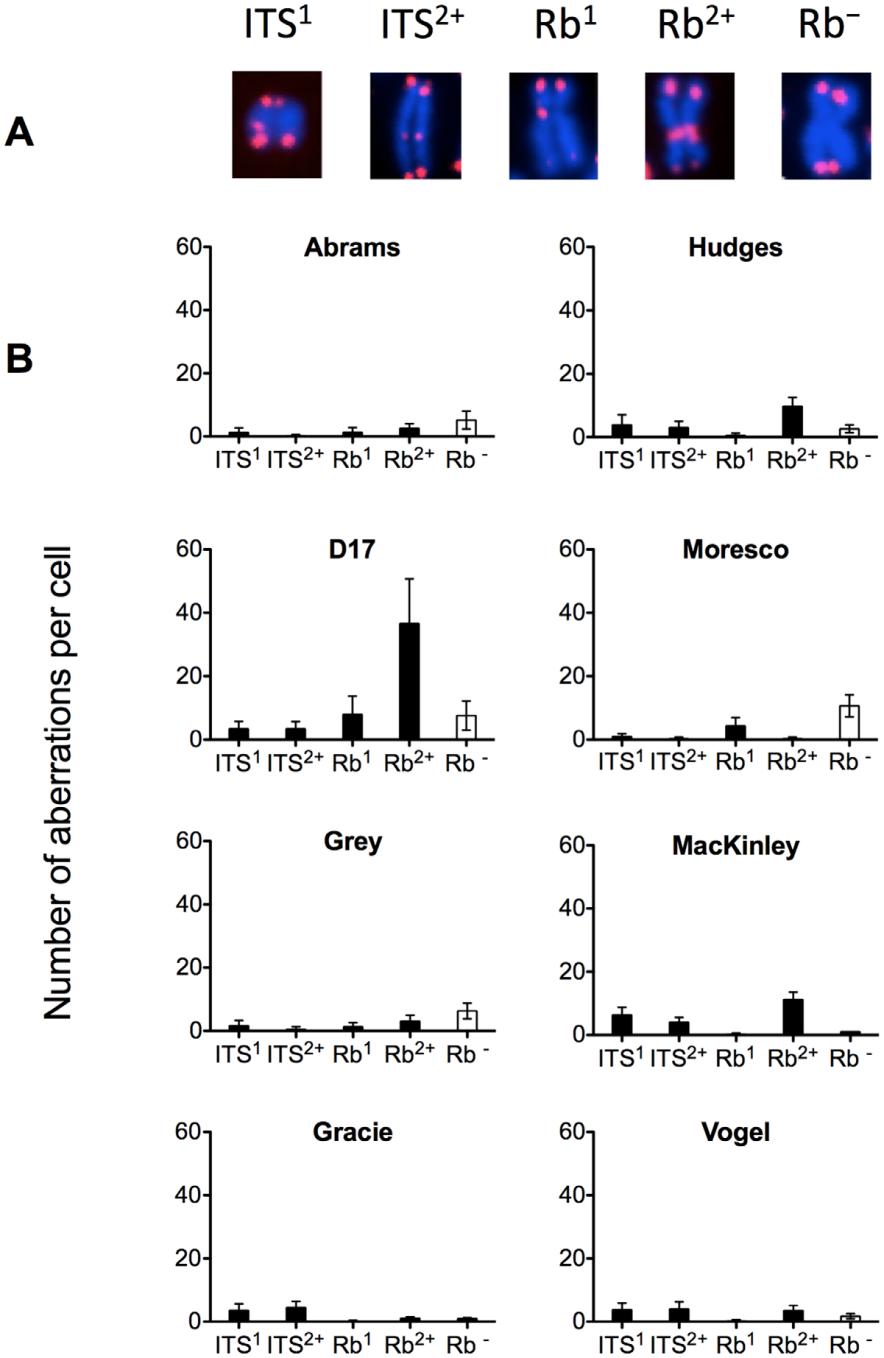
Telomere abnormalities distinguished by Rb fusions and interstitial signals in OSA cells. (A) Four types of telomere abnormalities; ITS^1^, one interstitial telomeric sequence, ITS^2+^, more than one interstitial telomeric sequences, Rb^1^, Robertsonian translocation with one telomere signal in the centromere region, and Rb^2+^, Robertsonian translocation with more than one telomere signals in the centromere region. Rb^−^ represents Robertsonian translocation with no telomere signal in the centromere region. (B) The number of telomere aberrations per each metaphase cell. Error bars indicate the standard error of the means.

Fluorescence *in situ* hybridization (FISH) with a PNA telomere probe revealed that all eight canine OSA cell lines exhibited numerous telomere fusions and interstitial telomere signals (Figure 3). Frequency distribution plots regarding the four types of telomere abnormalities in the eight cell lines is presented in Figure 4. Notable differences in the distribution of telomere fusion types were observed within the eight OSA cell lines. In this analysis, maximal telomere fusions were 36 per cell for D17 representing a Rb^2+^ translocation. D17, Hughes and MacKinley lines were also characterized by the Rb^2+^ telomere fusions, while Gracie and Vogel tended to have ITS^1^ and ITS^2+^ translocations. The numbers of telomere fusions in Abrams, Grey and Moresco lines were small, with these cell lines primarily exhibiting a Rb^-^, Robertsonian translocation with no telomere signal in centromeres. We observed different strength of telomeric signals between fusion points and regular telomeric ends of chromosomes. In D17, Hughes, Moresco, and MacKinley, the telomere signals were stronger in fusion points than in telomeric ends (Table 2); however, those values in fusion points was less than two.

**Table 2:** Summary of telomere abnormalities and other telomere associated factors in eight canine OSA cell lines.

OSA Cell line	No. ofColocalizations*	Sum of TelomereAbnormalities**	Signal Ratio ofTelomeres***	DNA-PKExpression****	TelomeraseActivity
Abrams	3.15±4.6	5.4±2.6	0.71±0.36	1.55	Positive
D17	3.26±4.4	51.4±17.5	1.12±0.63	2.88	Positive
Grey	4.82±4.4	6.4±3.2	0.92±0.38	0.60	Positive
Gracie	2.80±2.8	9.1±3.1	0.89±0.41	0.68	Positive
Hughes	3.10±3.1	17.2±5.7	1.61±0.96	0.20	Positive
Moresco	2.27±2.8	10.0±3.7	1.48±0.89	0.73	Positive
MacKinley	1.76±2.0	17.5±3.3	1.20±0.85	1.22	Positive
Vogel	1.48±1.6	11.4±3.5	0.83±0.35	0.13	Positive

* Mean ± SD of numbers per cell from more than 50 cells.

** Sum of four types of telomere abnormalities ± SD per cell from more than 30 cells.

*** Telomere signal ratio (at fusion area/at telomere area) ± SD.

**** The values are arbitrary unit. Average value of 8 cell lines is 1.

### Telomere and γ-H2AX Colocalization

**Figure 5:**
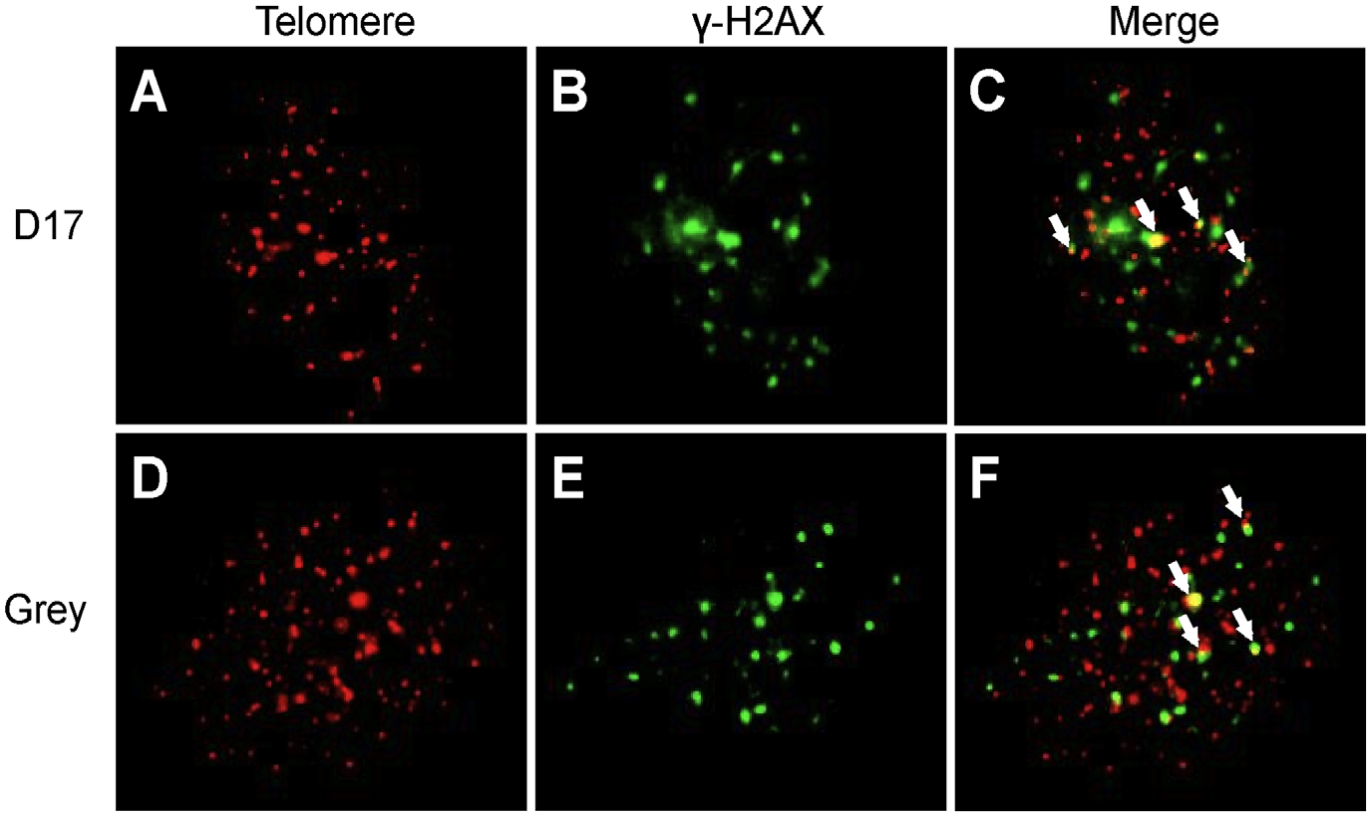
Representative images for colocalization of telomere signals and γ-H2AX foci in interphase nuclei of OSA cells. The D17 cell line is shown in panel A, B and C. D17 shows telomere signals (A) and γ-H2AX (B) and the merged image (C). (D, E and F) represent interphase nuclei of the Grey cell line. Arrows denote colocalizations.

To assess whether telomere aberrations were elevated in nuclear foci of phosphorylated H2AX resulting from DNA damage, we utilized γ-H2AX and FISH to assess colocalization. Specifically, we focused on measuring this type of damage in telomere regions of chromosomes. Figure 5 presents the colocalization of telomere signals and γ-H2AX foci in interphase nuclei. In each canine OSA cell line, the average numbers of colocalizations were approximately 1.5 to 4.8 per nucleus (Table 2). The appearance of the colocalization of telomere signals and γ-H2AX foci clearly shows DNA damage associated with these telomere fusions, and the canine OSA cell lines have unstable telomere.

### DNA-PKcs Protein Expression in Canine OSA

**Figure 6:**
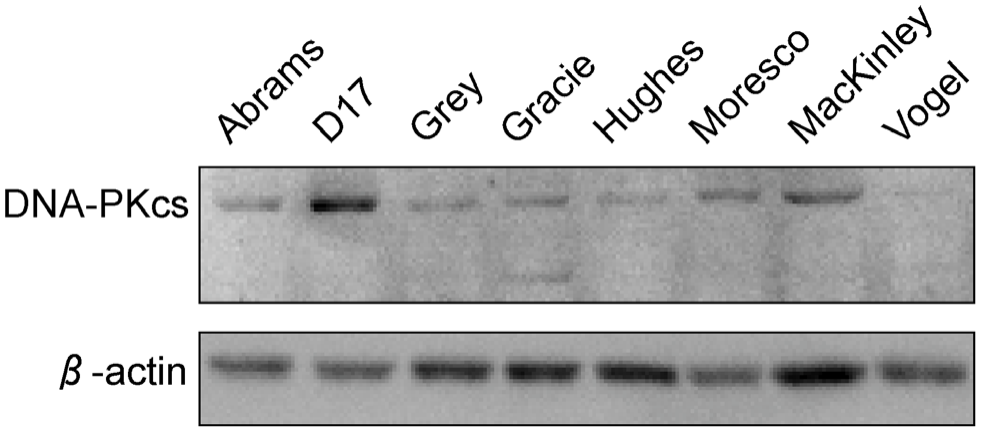
Western blot analysis of DNA-PKcs in the eight canine OSA cells. β-actin expression was used as a normalization control. DNA-PKcs is estimated from molecular weight (460 kDa).

We measured DNA-PKcs expression by western blot analysis (Figure 6). Expressions of DNA-PKcs were not uniform among the cell lines. We observed less expression of DNA-PKcs protein in Hughes (20% of average) and Vogel (12% of average) cells. The expression of DNA-PKcs in D17 was approximately three times more than the average of the 8 canine OSA cell lines. Abrams and MacKinley showed 50% and 20% more expression of DNA-PKcs compared to the average of 8 cell lines.

### Telomerase Activity by TRAP Assay

**Figure 7:**
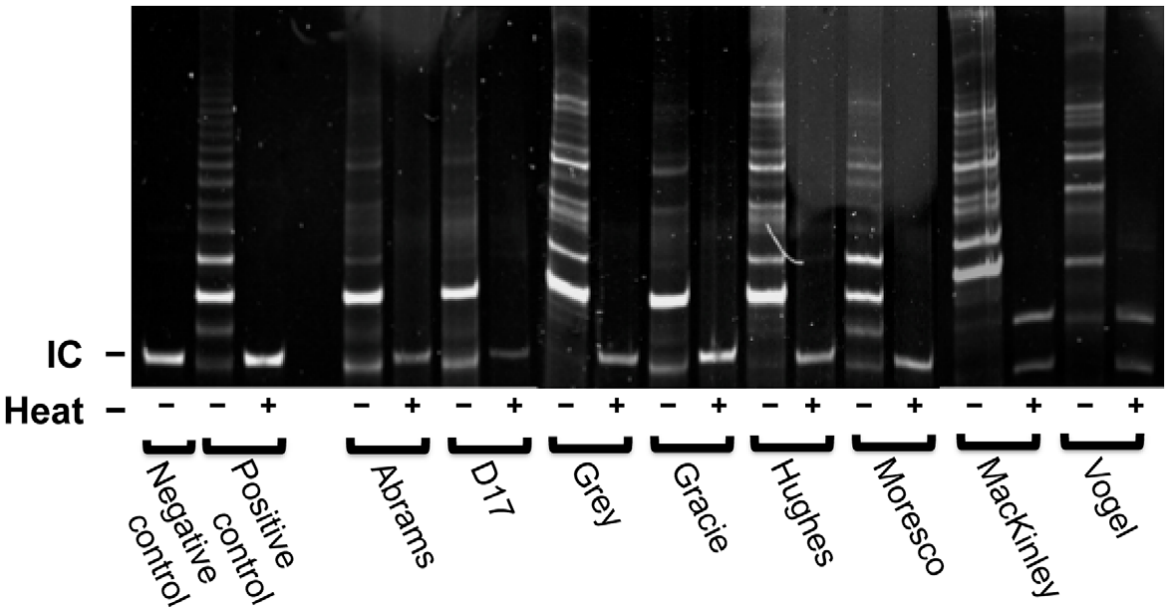
Telomerase activity in canine OSA cell lines. TRAP assay confirmed all cell lines expressed enzymatically active TERT. Positive controls were provided by the manufacturer. (−) Non–heated extract, (+) heated extract, IC: internal PCR control.

To further investigate the involvement of telomere fusion, we measured telomerase activity by TRAP assay in canine OSA cell lines. All eight of the OSA cell lines were determined to be telomerase positive. Heat inactivation was used for negative controls of each sample for telomerase activity. (Figure 7).

### Telomere Fusions in Spontaneous Canine OSA

**Figure 8:**
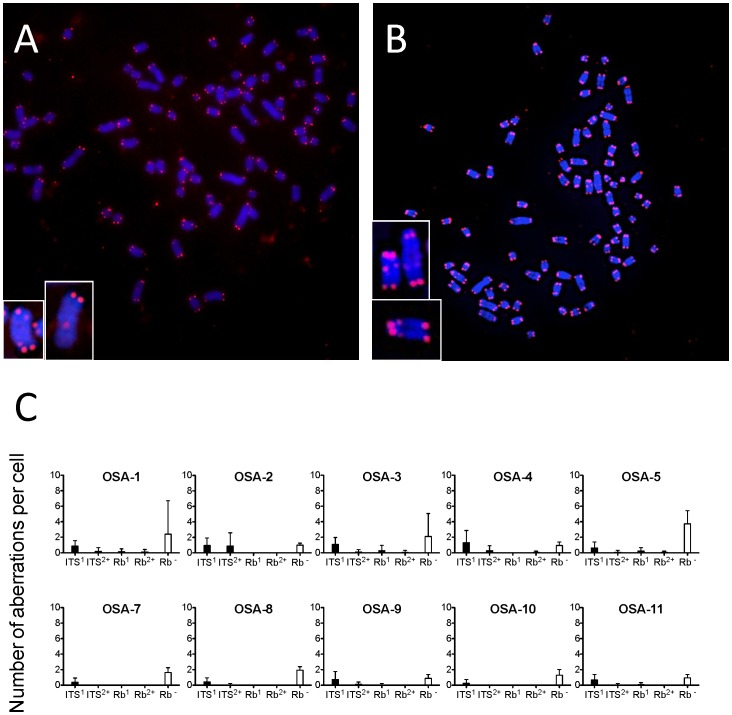
Primary canine OSA cell cultures and telomere fusions. Representative FISH images of the two primary canine OSA cell cultures’ metaphase chromosomes hybridized with probes against telomeres; OSA-1 (A), the sample originated from the limb, OSA-2 (B), the sample originated from the scapula. Note the abnormal telomere signals in the magnification box. Blue represents DNA staining by DAPI and red represents a telomere signal by Cy3. (C) The number of telomere aberrations per each metaphase cell. Error bars indicate the standard error of the means.

To eliminate a concern with the long-term culture effects, we analyzed telomere fusions in two primary canine OSA cell cultures, derived from tumors arising from the limb and scapular regions in 10 separate patients, and followed by cytogenetic analysis (Table 3). FISH with a PNA telomere probe revealed that the 10 samples of naturally occurring primary OSA exhibited abnormal telomeres as well as the eight established cell lines tested (Figure 8). Interestingly seven of the samples (OSA-2, OSA-4, OSA-7, OSA-8, OSA-9, OSA-10, and OSA-11) were with no increase of metacentric chromosomes, while three of the samples (OSA-1, OSA-3, and OSA-5) had increased numbers of metacentric chromosomes. The numbers of telomere fusions in the primary cultures were small with dominant ITS translocations.

**Table 3:** Sum of telomere abnormalities in ten primary canine OSA cell culture.

	OSA-1	OSA-2	OSA-3	OSA-4	OSA-5	OSA-7	OSA-8	OSA-9	OSA-10	OSA-11
No. of Chromosomes*	96.5±31.6	76.8±6.25	73.6±9.47	71.9±8.86	115.2±4.02	66.5±12.89	83.2±25.38	74.8±20.49	74.9±10.33	82.7±23.0
Sum of TelomereAbnormalities**	6.4±7.4	1.87±2.3	1.50±1.25	1.60±1.87	0.97±0.96	0.37±0.56	0.47±0.51	0.87±1.08	0.23±0.50	0.77±0.76

* Mean ± SD of chromosome number per cell from more than 100 metaphases.

** Sum of four types of telomere abnormalities ± SD per cell from more than 30 cells.

## Discussion

As observed previously in published studies using canine OSA cells [35], the eight canine OSA cells examined in this study possessed chaotic karyotypes comprising a wide range of both chromosome numbers and abnormal structures (Figure 1 and Table 1). Aneuploidy, hyperploidy and hypodiploidy of canine OSA from direct tissue biopsies has also been reported [36], [37]. In a previous study utilizing radiation-induced canine OSA cell lines, chromosome numbers presented with predominant ranges of 45 to 55 and 90 to 105 [14]. Furthermore, ranges of 10–15 and 20–30 abnormal metacentric chromosomes were observed in these cell lines [14]. Our results in the classical cytogenetic assay showed the established canine OSA to have remarkably different karyotypes clearly distinguishable from normal canine cell lines that confirms data found in previous studies (Table 1). Chromosome number instabilities were different among the eight canine OSA cell lines. Cell lines with bimodal peaks representing chromosome numbers displayed a wide range of chromosome numbers from cell to cell. Metacentric chromosomes have been shown to be a centric fusion for canine OSA cells [14], [36], and our results support this work. The frequencies of metacentric chromosomes in our eight canine OSA cell lines varied from higher to lower relative to findings of previous studies.

Several studies have addressed the high resistance canine OSA cells have towards radiation. In a previous study, the mean SF2 was relatively high (0.62), and the mean SF2 did not differ significantly among the four cell lines tested at these radiation doses [38]. Additionally, other studies in human tumor cell lines showed that cells with multiple copies of chromosomes tended to be resistant to ionizing radiation [39]. Contrary to these findings, we found both radioresistant and highly radiosensitive cell lines (Figure 2). Furthermore, the cell lines Grey and Hughes were shown to be the most sensitive to ionizing radiation while their average chromosome number were the highest among the eight cell lines.

The cell proliferation rates of canine OSA cell lines were vastly different from those previously reported [40]. We did not find any correlation between radiation sensitivities and cellular proliferation rates (Table 1), and the eight OSA cell lines utilized were varied in terms of chromosome numbers, radiosensitivity and cell proliferation.

Perhaps the most important finding in this study was the demonstration that telomere fusions are a potential causative factor regarding genomic instability in canine OSA (Figure 3). It is widely supported that telomere dysfunction could possibly play a causal role in early carcinogenesis through instigating a bridge-breakage fusion type chromosomal instability, leading to the promotion of neoplastic transformation [41]. Furthermore, the conversion from normal to aberrant karyotype via telomeric fusions has been reported using SV-40 transfection [34]. Although metacentric chromosomes have been shown and hypothesized to be a result from telomere fusions, our study identifies telomere fusion as a novel chromosome dysfunction in canine OSA cells.

We analyzed four telomere fusions including ITS and Rb (Figure 4). Through counting metacentric chromosomes and telomere abnormalities, we found further chaotic karyotypes among the eight canine OSA cell lines. Interestingly, telomere signals at the centromere region of metacentric chromosomes were not present in all cells with metacentric chromosomes except sex chromosomes. We observed that telomere signals at fusion points were less than twice the strength of telomere signals at the chromosome ends (Table 2). These results may support previous studies reporting that loss of telomeres leads to telocentric fusion resulting from two chromosomes with terminal deletions fusing to create one centromere fragment with less than four telomeric signals to avoid too short telomeres [42]. We confirmed that the telomere fusions also occurred in short-term cultures from 10 canine OSA clinical samples (Figure 8 and Table 3). Based on the observation of telomere aberrations in these primary cultures, we are reasonably certain that we are indeed working with an *in vivo* situation and not genomic alteration due to prolonged culture. In the 10 primary OSA cell cultures tested, frequency distribution plots regarding the four types of telomere abnormalities is less than in the eight canine OSA cell lines. These results appeared telomere fusions are clear characteristics of canine OSA but enhanced by long passages.

We observed colocalization of γ-H2AX and telomere signals in interphase cells (Figure 5). Nuclear foci of phosphorylated H2AX are sensitive markers for DNA double-strand breaks (DSBs) [43]. Analysis of γ-H2AX foci in different cultured tumor cell lines has shown that DSB damage plays a prominent role in the instability of telomeres in tumor cells [44], [45], and our results support the idea that DNA damages or shortening of telomeres may also lead to elevated numbers of telomere fusions [46].

Western blotting analysis suggested that these eight cell lines have different levels of expression of DNA-PKcs (Figure 6). DNA-PKcs is one of telomere maintenance proteins preventing telomere fusion [32]. We observed that high expression of DNA-PKcs in D17 cells and low expression in Vogel cells. D17 cells showed highly chaotic karyotypes and high amounts of telomere fusion. Our results suggested that reduction of DNA-PKcs is not always the cause of telomere fusion in canine OSA cell lines. Other proteins, many of which are commonly associated with DNA repair, are also required for effective telomere protection [47]. Altered function of other telomere maintenance proteins including TRF2, RAP1, and POT1 might be related to telomere fusions in canine OSA [48], [49], [50].

We attempted to correlate telomere fusions and other characteristics associated with classic cytogenetic analysis, radiosensitivity and DNA repair, but were unable to establish any significant correlations. The eight canine OSA cell lines utilized in our study, which all exhibited altered telomere signals, were all from cells which were telomerase positive contrary to a previous study describing 27% of canine OSA samples presenting as telomerase negative [22]. These results suggest the requirement of further exploration regarding telomere fusion in telomerase negative canine OSA cells. Moreover, metacentric chromosomes have been shown to be a centric fusion for other solid tumors, such as transmissible sarcoma, hemangiopericytoma, hemangioendothelioma, spindle-cell sarcoma, mammary carcinoma, and cutaneous mast cell tumors in dogs [51], [52], [53], [54]. Our study suggests that telomere fusions might be a significant diagnostic marker and potential treatment target proceeding further research for canine tumors.

In conclusion, we tested eight canine OSA cell lines that have different karyotypes, radiation sensitivity, and proliferation rate for cytogenetic analysis. All cell lines and primary cell cultures we tested showed telomere fusions. Pending further research regarding telomerase negative canine OSA cell lines, our findings suggest telomere fusions may be a novel marker for canine OSA.

## Materials and Methods

### Cell Lines and Culture

The canine OSA cell lines Abrams, D17, Grey, Hughes, and Moresco and human OSA cell line U2OS were supplied as previously described [40], and Gracie, MacKinley, and Vogel were kindly supplied by Animal Cancer Center of Colorado State University (Fort Collins, CO, USA). All OSA cell lines were grown in Minimum Essential Medium (MEM/EBSS, Thermo Fisher Scientific, Waltham, MA) supplemented with 10% fetal bovine serum (FBS; Sigma-Aldrich, St Louis, MO), 1% MEM vitamins, non-essential amino acids, sodium pyruvate, penicillin, streptomycin and fungizone. Cell lines were maintained at 37°C, humidified with 5% CO_2_.

### Primary Canine OSA Cell Culture Preparation

Ten tumor samples from dogs diagnosed with OSA, presenting with disease limited to the limb and the scapular region, were collected under an approved Institutional Animal Care and Use Committee protocol with informed owner consent. Dissected tumor samples were collected immediately after surgery, treated with collagenase for three hours and cultured in Minimum Essential Medium with 15% fetal bovine serum, 1% MEM vitamins, non-essential amino acids, sodium pyruvate, penicillin, streptomycin and fungizone. Cell cultures were maintained at 37°C, humidified with 5% CO_2_. Experiments were carried out using less than three passage cell cultures.

### Chromosome Number

Cells were cultured with 0.1 µg/mL colcemid (GIBCO, Invitrogen, Carlsbad, CA) for six hours in order to harvest metaphase chromosomes. Samples were treated in hypotonic 75 mM KCl solution for 20 minutes at 37°C and fixed in 3∶1 (methanol: acetic acid) fixation solution three times. Spread metaphase chromosomes were stained with Giemsa solution, and the chromosome number was observed under a BX51 microscope (Olympus, Tokyo, Japan). A minimum of 150 metaphase cells were analyzed for two separate experiments. At least 75 metaphase cells were analyzed to count metacentric chromosomes per cell. For primary cell cultures, 100 metaphase cells were analyzed for a single experiment.

### Cell Proliferation

In order to determine the carrying proliferation rates of the cell lines, a five-day proliferation trial was performed. Five thousand cells were plated in T12.5 flasks. The number of cells was counted every 24 hours for five days by coulter counter (Beckman Coulter, Brea, CA). Cellular doubling times were calculated by GraphPad Prism 5 software.

### Irradiation and Cell Survival

Randomly dividing log phase cultures were irradiated with ^137^Cs gamma-rays delivered at a dose rate of approximately 2.5 Gy per minute at room temperature (using a J.L. Shepherd Model Mark I-68 6000Ci ^137^Cs irradiator). Sensitivity to radiation was evaluated by colony formation assays. Cells were exposed to ionizing radiation, treated with trypsin-EDTA and plated onto 100 mm culture dishes at appropriate cell density. After incubating for 7–14 days to allow colony formation, surviving colonies were rinsed with 0.9% NaCl, fixed with 100% ethanol and stained by 0.1% crystal violet. Each colony consisting of more than 50 cells was scored as a survivor. At least three independent experiments were carried out.

### Flow Cytometry

Cells were trypsinized, washed once with PBS and fixed in 70% ethanol. The fixed cells were collected by centrifugation and resuspended in 20 µg/mL propidium iodide and 500 µg/mL RNase A. The DNA contents were measured using FacsCalibur Flow Cytometer and the Cell Quest Pro program (BD Biosciences, Franklin Lakes, NJ). The ploidy levels of the eight canine OSA and CHO (Chinese hamster ovary) cells were defined by the DNA peak value of the cells. CHO cells were used as the control of diploidy. Each cell lines were gated at 10,000 events via the flow cytometer.

### Fluorescence In Situ Hybridization (FISH) for Telomeres

Cells were synchronized in metaphase by 0.1 µg/mL colcemid treatment. Samples were incubated at 37°C in a hypotonic solution of 75 mM KCl for 20 minutes and fixed three times in 3∶1 (methanol: acetic acid) fixation solution. Cells dropped on slides were treated with 100 µg/mL RNAse A for ten minutes at 37°C, fixed in 4% formaldehyde, and rinsed in PBS. The slides were denatured by 70% formamide/2×Saline sodium citrate (SCC) buffer (3 M NaCl, 0. 3 M sodium citrate, pH 7.0) at 75°C for two minutes, followed by dehydration in ethanol series. Peptide-nucleic acid (PNA) telomere probes (DAKO, Carpinteria, CA) were denatured at 75°C for 5 minutes. The denatured probes were added to the fixed cells on slides and kept in a humidified dark chamber at 37°C for three hours. Slides were then washed in 70% formamide/2×Saline sodium citrate (SCC) buffer at 32°C for 15 minutes and in Sodium phosphate (PN) buffer (0.1 M NaH_2_PO_4_, 0.1 M Na_2_HPO_4_, pH 8.0 and 0.1% NP40) for five minutes. Lastly, slides were counterstained with DAPI, and photographed using a BX61 microscope and a cooled CCD Exi Aqua camera (Q-imaging, BC, Canada).

### Telomere Fusions

The telomere fusions of a minimum of 27 metaphase cells were scored for the presence of marker aberrations. Four types of telomere fusions; ITS^1^, a single interstitial telomeric sequence, ITS^2+^, multiple interstitial telomeric sequences, Rb^1^, Robertsonian translocation with a telomere signal in the centromere, and Rb^2+^, Robertsonian translocation distinguished by more than one telomere signal in centromere region were used for telomere fusion scoring. The telomere signal strength was measured by the line measurement function of Q-capture Pro software (Q-imaging). The diameter of telomeric signal was measured and the mean ratio (fusion points/chromosome ends) was obtained. At least 30 fusion points and 100 chromosome ends were analyzed.

### Immuno-telomere FISH with Phosphorylated H2AX Immunocytochemistry

Cells were cultured for 24 hours on plastic chamber slides and then washed with PBS followed by fixation with 4% paraformaldehyde for 15 minutes. Following a second wash with PBS, cells were then permeabilized with 0.2% Triton X 100 in PBS for ten minutes. Cells were then blocked in PBS with 10% goat serum overnight at 4°C. Following overnight incubation, the cells were incubated with a mouse monoclonal phosphorylated histone H2AX antibody (Ser139) (Millipore, Billerica, MA) in 10% goat serum with PBS for one hour at 37°C. The cells were then washed three times for ten minutes each in PBS, followed by incubation for one hour at 37°C with Alexa 488 Fluor-conjugated goat anti-mouse antibody (Molecular Probes, Eugene, OR). The slides were then washed three times for ten minutes each in PBS. After 15 min paraformaldehyde treatment, PNA-FISH was carried out as described above. The cells were cover slipped and visualized with Olympus BX51 equipped with a cooled CCD Exi Aqua camera (Q-imaging). Q-CapturePro was utilized to obtain images. Numbers of the colocalizations of telomere signals and H2AX were counted for a minimum of 50 cells.

### Telomerase Activity

Telomerase activity was measured by the commercially available TRAPeze® Telomerase Detection Kit (Millipore) according to the manufacture’s instructions with the below mentioned minor modifications. After further incubation at 30°C for 30 minutes, an additional step was held at 90°C for three minutes. The resulting mixture was subjected to PCR for 34 cycles of 30 seconds at 94°C, 30 seconds at 59°C, and one minute at 72°C. Final elongation was performed at 72°C for three minutes. PCR products were run on NOVEX 15% nondenaturing TBE-PAGE gels (Invitrogen), stained with 1∶10,000 ethidium bromide in deionized water for 30 minutes, and destained in deionized water for an additional 30 minutes at room temperature. Visualization of PCR products was performed with a ChemiDoc™ XRS Imager (Bio-Rad, Hercules, CA).

### Western Blotting

Cells were lysed with M-PER Mammalian Protein Extraction Reagent (Thermo Fisher Scientific) and protease inhibitors, Halt Protease Inhibitor Cocktail Kit (Thermo Fisher Scientific). Protein extracts (20 µg per sample) were size-fractionated on NuPage® 4–12% Bis-Tris gels (Invitrogen), electro-transferred to nitrocellulose membranes (Bio-Rad) in a buffer (25 mM Tris, 192 mM glycine, 20% (v/v) methanol, and 0.01% SDS) at a current density of 3.0 mA/cm^2^ for 16 hours at 4°C. The filters were blocked with Tris-bufferd saline with 0.05% Tween 20 containing 2% (w/v) skim milk, and reacted with the mouse anti-DNA-PKcs monoclonal antibody (Ab-4; Neomarkers, Fremonti, CA) (1∶1000), followed by an incubation with goat anti-mouse IgG HRP-conjugated antibody (1∶10,000) (Santa Cruz Biotechnology, Inc., Santa Cruz, CA). The immunoreactive signals were detected by using SuperSignal Western Blotting Detection Kit (Thermo Fisher Scientific) and ChemiDoc™ XRS+System (Bio-Rad). Protein expression from band strength was analyzed by Image Lab software (Bio-Rad).

### Statistical Methods

Analysis of variance was used to determine statistical significance with GraphPad Prism 5 software (Graph Pad Software, La Jolla, CA). For all analyses, P values of less than 0.05 were considered statistically significant.
